# Newspaper Notices of Medicines, Medical Men and Quacks

**Published:** 1871-06

**Authors:** 


### Editorial.

### Newspaper Notices of Medicines, Medical Men and Quacks.

The newspaper press furnishes the great majority of families in our country with the principal part of their reading. When the morning paper has been read, other duties claim attention, and nothing more is taken up until the arrival of the next issue of their accustomed sheet. The chosen paper is a kind of law to many, it is the exponent of their political or christian faith, and in many cases, it is also their standard authority in medical belief. The popular press has been mainly subsidized to the interests of quackery, and imposition. Regular medicine has thus far required little *advertising*, while imposters in medicine, and worthless and inert compounds have afforded revenues to publishers which have been important, and thus far, controlling. The influence of this advertising over the masses of mankind is truly astonishing, so much so, that the dupes themselves, conscious of the folly, ridicule and curse, and then accept as "better than advertised." Itinerant quacks herald their approach, and their paid announcements are made to read as though the editor was the father of them; they would thus father Satan, for due consideration and sometimes, without much pay, we mistrust. The political and secular press, give large space to abominable falsehood, every word of which is well known to be a lie; paid for, of course, but none the less a lie. Our worthy editors are very estimable citizens as editors. They furnish much knowledge of this world's working, and we could not do without them, no, not at all. We entertain for them profound respect as *editors*, but they show that they have never studied medicine, and are wholly incapable of giving the public any rational knowledge of medicine.

The religious press, too, attempts to teach everything; one must read with considerable attention to determine what he is perusing, his difficulty being to decide if he has a religious tract, a political speech, an Almanac or a Patent Medicine circular. If they are not more correct in their religious teaching than in medicine, they will have their part with the great, great grandfather of all lies, and that is the only disposition that can be made of them. We do not propose to organize any society to correct the customs of our editorial friends in this respect, but expect they will continue to do that which pays them best. We only desired to state the general facts, and leave it as the history of our times, and country and profession.

It does not stop here. The issues of the daily press contain mention of worthy physicians, in the same language, and apparent style, in which they speak of the quacks. It has come to be supposed that physicians who do not write or seek such notice, are yet glad to receive it, if placed gratuitously in connection with the reporters account of death or attempted suicide or murder or surgical operation, or something else, in which noble and praiseworthy deeds may be announced.

A very intimate friend of ours was visited a day or two since by a countryman, who had slight injury of the hand. He says, "is this doctor——," yes, sir, that is my name, was the reply. "Well, I see your name mentioned in the newspapers pretty often in taking care of such things, and have come some ways to get you to just look at my hand." The laconic reply was, "I am not the man spoken of at all, and if one of these editors ever speak of me in connection with accidents or injuries, I will give him a notice which shall silence his quill forever. Rage and grief still agitating him, he proceeded immediately to the editorial sanctum and requested the discontinuance of such practice. Mr. Editor replied, in astonishment. "We can, generally, leave out *your* name, but these items come to us with the *names in*—the *name* being the most important point in the item.

It is useless, in the present debauched state of popular journalism, so far as Medicine, Quack Doctors and real Physicians are concerned, to expect any great change, but it is not very unreasonable to suppose, that in most places, persons injured can obtain satisfactory care, and if some one has broken a leg, it is not an important point in the case to state by what physician it was dressed. With my friend, I will forgive everything in the public press if it will keep silence in regard to the operations of physicians. May do anything and say anything of Quack Doctors and Quack medicines they please, may spread religion and politics and all healing Balsams and Strengthening plasters, eternally upon their sheets if they can make any money out of it, and we will not complain of personal abuse. As a journalist, it will, however, remain an open question, upon which many an editorial may be based, always protesting against the mention of an honest physician in the advertising mediums of the Quacks.

